# Maternal and paternal height and BMI and patterns of fetal growth: The Pune Maternal Nutrition Study

**DOI:** 10.1016/j.earlhumdev.2010.07.002

**Published:** 2010-09

**Authors:** Andrew K. Wills, Manoj C. Chinchwadkar, Charudatta V. Joglekar, Asit S. Natekar, Chittaranjan S. Yajnik, Caroline H.D. Fall, Arun S. Kinare

**Affiliations:** a MRC Epidemiology Resource Centre, University of Southampton, UK; b MRC Unit for Lifelong Health and Ageing, Department of Epidemiology and Public Health, University College London, UK; c KEM Hospital Research Centre, Pune, India

**Keywords:** Fetal growth, Parental determinants, Intrauterine effects, Placenta, Body mass index

## Abstract

We examined the differential associations of each parent's height and BMI with fetal growth, and examined the pattern of the associations through gestation. Data are from 557 term pregnancies in the Pune Maternal Nutrition Study. Size and conditional growth outcomes from 17 to 29 weeks to birth were derived from ultrasound and birth measures of head circumference, abdominal circumference, femur length and placental volume (at 17 weeks only). Parental height was positively associated with fetal head circumference and femur length. The associations with paternal height were detectible earlier in gestation (17–29 weeks) compared to the associations with maternal height. Fetuses of mothers with a higher BMI had a smaller mean head circumference at 17 weeks, but caught up to have larger head circumference at birth. Maternal but not paternal BMI, and paternal but not maternal height, were positively associated with placental volume. The opposing associations of placenta and fetal head growth with maternal BMI at 17 weeks could indicate prioritisation of early placental development, possibly as a strategy to facilitate growth in late gestation. This study has highlighted how the pattern of parental–fetal associations varies over gestation. Further follow-up will determine whether and how these variations in fetal/placental development relate to health in later life.

## 1 Introduction

Low birth weight and size are related to the risk of cardiovascular disease and type 2 diabetes later in life [1,2]. Part of this relationship is thought to be explained by developmental programming, which suggests that environmental exposures occurring during developmental periods cause physiological adaptations that alter the long term propensity for disease [3,4]. In this sense, fetal growth is a marker of a disturbed prenatal environment. Investigating environmental and genetic determinants of fetal growth may enhance our understanding of the associations between birth size and later life health. And from a public health perspective, intrauterine effects due to modifiable maternal factors, such as diet or adiposity, are crucial as they provide additional opportunities for intervention. This has particular relevance in a developing country such as India where the prevalence of low birth weight is as high as 30% [5].

Maternal and paternal height and body mass index (BMI) capture information on the genetic potential of the offspring, the shared environment of the parents, and the historical and present nutritional condition of the mother. The latter is reflected onto the fetus' own nutritional status in what we refer to as intrauterine effects. There is evidence that parental anthropometry is strongly associated with birth size. For example, maternal BMI and height explain a large proportion of the geographical variation in birth weight, length and head circumference [6], and paternal height is associated with birth weight [7–9] and length [8], independent of maternal height. One way of disentangling intrauterine effects from the inherited component of size and growth is to compare the size of the association between the mother and offspring with the size of the association between the father and offspring [10,11]. If intrauterine effects are present, then we would expect the maternal associations to be stronger than the paternal association. Previous studies showed that the height of both parents correlate approximately equally with newborn length, while maternal BMI correlates more strongly than paternal BMI with measures of newborn soft tissue mass [6].

A limitation of birth anthropometry as a proxy for fetal growth is that two babies of a similar size at birth may have had radically different growth trajectories in-utero [12]. The absence of an association between a maternal exposure and birth size therefore, does not necessarily exclude an effect acting during a period of fetal development. Serial fetal ultrasound alongside data from birth should provide a better marker of growth restriction. Fetal growth is characterised by substantial centile crossing in individual growth trajectories suggesting it is a highly adaptive process [13]. Studies to date have tended to examine cross sectional associations at each time of measurement [14] or modeled the trajectory using multilevel methods [15,16]. An alternative which may capture this adaptive process more directly is to examine growth conditional on earlier size, this approach allows growth at different periods of gestation to be examined independently of earlier size and growth, and thus an examination of the onset and pattern of associations over the course of gestation.

Data from mother–father–fetus/offspring trios from the Pune Maternal Nutrition study were used to: [1] examine the association between parental height and BMI and fetal head circumference, abdominal circumference and femur length, [2] assess the relative influence from the mother and father, and [3] explore the timing and behavior of these associations over gestation. In an *a posteriori* analysis, we also examined the relationship with placental volume at 17 weeks gestation.

## 2 Methods

### 2.1 Study population

Data are from the Pune Maternal Nutrition Study (PMNS), a rural Indian community-based prospective study. The cohort was established from a house-to house survey of all married women of childbearing age (15–40 years) living in 6 villages located 40–50 km from Pune City (n = 2675). Enrolment took place between 1994 and 1996; 2466 women (92%) agreed to participate and 1102 became pregnant. The majority of women were vegetarian and had below recommended intakes of energy and protein. Further details are elsewhere [17].

### 2.2 Data collection

#### 2.2.1 Parental data

Each woman's height and weight were measured by health workers every three months until pregnancy occurred; the last set of measurements prior to conception was used as pre-pregnant values. Paternal height and weight were also measured within 3 months of the confirmation of the woman's pregnancy. BMI was calculated as wt/ht^2^.

Several potential confounding variables were examined. Information on household socio-economic status was collected using a standardised questionnaire [18], this derives a composite score based on the occupation and education of the head of the household, caste, type of housing, and family ownership of animals, land and material possessions (5 level ordinal scale). Maternal parity and religion were recorded. None of the women were smokers, but it was recorded if there were any other smokers in the household (Yes/No).

#### 2.2.2 Fetal ultrasonography and birth measures

Each woman was visited monthly by a trained health worker to record the date of the last menstrual period (LMP). Women who reported a missed period underwent an ultrasound examination 15–18 weeks after their LMP to confirm pregnancy. A further ultrasound scan was scheduled for 28 ± 2 weeks gestation. The median gestational age at each examination was 17.1 (IQR: 16.6, 18.0) and 29.4 (IQR: 28.6, 30.1) weeks.

On each visit, one of two trained sonologists (MCC and ASK) obtained measures of fetal head circumference (HC), biparietal diameter (BPD), abdominal circumference (AC) and femur length (FL). Scans were carried out using a portable machine fitted with a curvilinear array 5 MHz transducer (ALOKA SSD 500, version 8.1, Osaka, Japan). Placental volume was measured at the first ultrasound scan only, with a linear-array 3.5 MHz transducer with a foot length of 14 cm and a 12.5 cm field of vision, using a modified planimetric technique [19]. The inter-observer variation in the extracted fetal measurements was excellent (0.004–0.04%). Full technical details on the fetal measurements [20] and the placental measurements [21] have been reported elsewhere.

Health workers performed detailed anthropometry of the babies within 72 hours of birth using standardised techniques. Birthweight was measured using a Salter spring balance; crown–heel length was measured using a portable Pedobaby Babymeter (ETS J.M.B., Brussels, Belgium); occipito-frontal head circumference and abdominal circumference were measured using a fibre glass tape (CMS Instruments, London UK), the latter immediately above the umbilical cord insertion, in expiration.

### 2.3 Gestational dating

For the purpose of this analysis, we used LMP dates to estimate gestation — this is important in a study of fetal growth, because ultrasound dating assigns a gestational age based on fetal size, thus erasing variation in growth during early pregnancy. However, to minimize errors from erroneous LMP dates, fetuses whose gestational age estimated by ultrasound differed by more than 2 weeks from the LMP estimate were excluded (n = 144). Sonographic gestational age was determined on the first visit, using a prediction equation based on fetal BPD, AC and FL [22].

### 2.4 Analysis sample

**Fig. 1:**
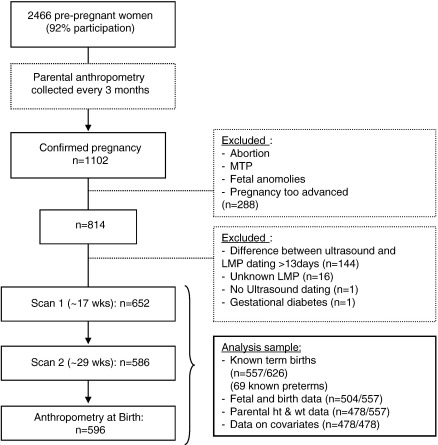
Participant and analysis flow chart.

From 1102 pregnancies, in addition to those excluded due to gestational dating discrepancies, 288 were excluded due to spontaneous abortions, fetal anomalies on ultrasound, multiple pregnancies, medical terminations or pregnancies detected later than 20 weeks gestation (Fig. 1). Babies born pre-term (< 37 weeks) were excluded because they may show different parent–offspring relationships due to underlying morbidity. One baby whose mother had gestational diabetes was also excluded. The final sample comprised 478 babies with complete parental size, ultrasound and birth data (Fig. 1).

### 2.5 Standardisation

Exposures and outcomes were standardised to a z-score so that we could compare the relative associations from the mother and father, and across fetal components. Parental BMI was log transformed before standardisation. Growth models were developed to describe the relationship between the fetal variables (HC, AC and FL) and gestational age using the method described by Royston (1995) [23]. These were used to compute z-scores at scan 1 and 2, and are reported elsewhere [13]. At birth, z-scores for head circumference, abdominal circumference and crown–heel length were estimated using ordinary regression accounting for gestational age and stratifying by sex, a similar method was used for placental volume at the 1st scan.

### 2.6 Analysis

#### 2.6.1 Conditional growth variables

Our analysis strategy was to assess associations of parental height and BMI with fetal size at scan 1 (17 weeks), then determine the association with growth from scan 1 to scan 2 (17–29 weeks) independent of size at 17 weeks, and move forward again to assess associations with growth from 29 weeks to birth independent of size at 17 and 29 weeks. To do this, we created 2 conditional growth variables for each fetal component — size at 29 weeks conditional on size at 17 weeks (29| 17 wks), and size at birth conditional on size at 29 and 17 weeks (Birth| 29 and 17 wks), by regressing each size measurement (z-score) on earlier size (stratified by sex) and keeping the standardised residuals. The conditional z-score is a measure of growth velocity between 2 time points (e.g. 17–29 weeks) on a theoretical distribution which compares each fetus against other cohort members of the same size at time point 1 (e.g. 17 weeks). It can be interpreted as growth above or below that expected given earlier size. The set of variables (size at 17 weeks; 29| 17 wks; Birth| 29 and 17 wks) thus contain information on whether a fetus grew quickly or slowly for the intervals 0–17, 17–29 and 29-birth relative to other fetuses of the same growth history. Importantly, by construction, the set of conditional variables are uncorrelated, and so any association between an exposure and an interval is independent of its association with other intervals.

#### 2.6.2 Statistical tests

The outcome variables were the growth set for HC, AC, FL and placental volume at 17 weeks. We had no data on FL or leg length at birth so used birth length as a proxy for birth femur length; these have been shown to be closely related [24]. We also estimated the association with overall size at 29 weeks and birth. Multivariable regression was used to examine the independent associations between parental height and BMI and the fetal outcomes. We pooled sexes as there was only one sex interaction out of 36. We adjusted for socio-economic status (SES), smoking and religion (Hindu/Buddhist, Muslim). Although there was little suggestion of confounding, we kept them in the models along with maternal age and parity because they increased the precision of the exposure estimates. Non-linear associations were assessed using plots and Wald tests of quadratic terms. We tested for a difference between the size of the mother–offspring association and the father–offspring association by reparameterising the model [25]. STATA v10 was used for all analyses.

#### 2.6.3 Misattributed paternity

False paternity will dilute the paternal offspring relationship and bias the comparisons of the mother–offspring and father–offspring relationship in favour of showing larger maternal effects. We performed a sensitivity analysis to examine the effect of non-paternity using a previously described method [26]. This method corrects the estimated coefficients for the mother and putative father by modifying the covariance matrix under the assumption that the non-biological father's BMI is unrelated to the offspring's BMI but is related to the mother's BMI to a similar magnitude of the biological father's BMI. To try to include the true non-paternity rate we simulated rates up to 15%.

## 3 Results

Mothers were short, light and thin (Table 1) — 65% were underweight (BMI < 18.5 kg/m^2^, WHO guidelines). Most were younger than 22 years and one-third were primiparous. Fathers were also short and thin (Table 1) — 39% were underweight. The correlations between maternal and paternal height and between maternal and paternal BMI were 0.19 and 0.13 respectively. The mean birth weight was 2597 g and 26% had a low birth weight (Table 1).

### 3.1 Parental height

**Fig. 2:**
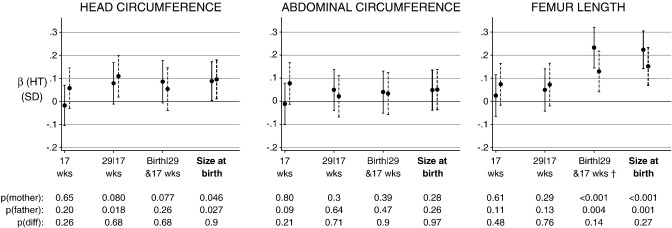
Standardised associations (95% CIs) of maternal (solid lines) and paternal (dashed lines) height with fetal size and growth at 17 weeks, 29|17 weeks and Birth|29 and 17 weeks and 95% CIs, and with overall size at birth (thick line). Adjusted for each other, maternal age, parity, SES, religion and household smokers. The p-values are for the mother and father, and for a test of the difference between the parent's coefficients. † Birth length conditional on FL at 29 and 17 weeks.

Maternal height was not related to fetal HC at 17 weeks (Fig. 2). There were positive associations with conditional growth between 17 and 29 wks, and between 29 wks and birth although the evidence was weak (p = 0.08 for both) (Fig. 2). At birth, an SD increase in maternal height (5 cm) was associated with a 0.09 SD increase in birth HC (Fig. 2).

There was no evidence for an association between paternal height and HC size at 17 weeks. Paternal height was positively related to growth in fetal HC between 17 and 29 weeks — an SD increase in the father's height (6 cm) was associated with a 0.11 SD increase in the part of HC growth from 17 to 29 weeks not associated with HC size at 17 weeks. There was no evidence for an additional effect of paternal height from 29 weeks beyond that which occurred earlier, however, paternal height was related to unconditional HC size at birth (Fig. 2).

There was no evidence of an association between maternal height and any AC growth or size parameters. There was a very weak suggestion of a positive association between paternal height and AC at 17 weeks. Paternal height was not related to AC in any of the other independent growth intervals or to AC size at 29 weeks or birth (Fig. 2).

There were no associations between maternal or paternal height and femur length (FL) at 17 weeks, or conditional growth from 17 to 29 weeks (Fig. 2). Both maternal and paternal height were positively related to length at birth conditional on earlier FL, and with overall length at birth. An SD increase in maternal and paternal height was associated with a 0.22 SD and 0.15 SD increase in the part of birth length not associated with femoral growth up to 29 weeks. Unlike maternal height, paternal height was related to unconditional FL size at 29 weeks (β (per SD) = 0.1SD, p = 0.024; data not shown in Fig. 2).

Despite a suggestion that the associations with paternal height were apparent earlier in gestation, there was no evidence for a difference between maternal–offspring and paternal–offspring relationships for any period of gestation or for any fetal component (Fig. 2).

### 3.2 Parental BMI

**Fig. 3:**
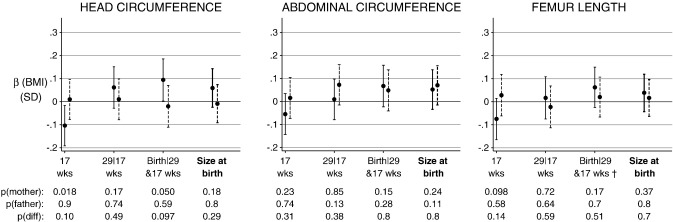
Standardised associations (95% CIs) of maternal (solid lines) and paternal (dashed lines) BMI with fetal size and growth at 17 weeks, 29|17 weeks and Birth|29 and 17 weeks, and with overall size at birth (thick line). Adjusted for each other, maternal age, parity, SES, religion and household smokers. The p-values are for the mother and father, and for a test of the difference between the parent's coefficients. † Birth length conditional on FL at 29 and 17 weeks.

Pre-pregnancy maternal BMI was negatively associated with HC size at 17 weeks (β (per SD) = − 0.11 SD; 95% CI: − 0.19, – 0.02) and positively associated with conditional HC growth from 29 weeks to birth (β (per SD) = − 0.09 SD; 95% CI: 0.0, 0.18) (Fig. 3). There was no evidence for an association between maternal BMI and fetal AC or FL size or growth (Fig. 3).

Paternal BMI did not appear to be related to any fetal growth or size component (Fig. 3), and despite there being some evidence for a maternal BMI–offspring relationship, tests of a difference between the parent's coefficients did not support differential parent effects.

### 3.3 Placental volume at 17 weeks

**Fig. 4:**
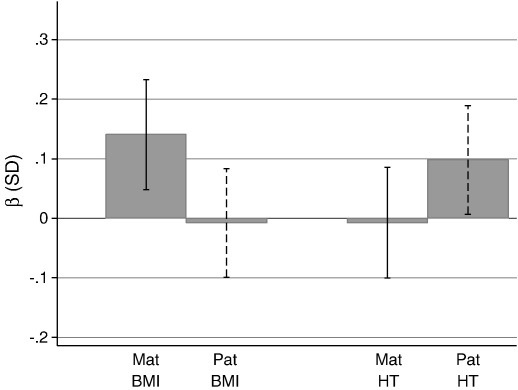
Standardised associations (95% CIs) with placental volume at 17 weeks gestation. Adjusted for each other, maternal age, parity, SES, religion and household smokers.

The median (IQR) placental volume at 17 weeks gestation was 148.6 ml (120.5, 179.2). Maternal BMI ((β (per SD increase) = 0.14 SD; p = 0.003) and paternal height ((β (per SD increase) = 0.1 SD; p = 0.02) were associated with placental volume at 17 weeks. There was no evidence for a relationship with maternal height or paternal BMI (Fig. 4). Placental volume was more strongly related to maternal BMI than paternal BMI (difference in β = 0.15, p = 0.034), there was no evidence that these associations differed for parental height (p for difference = 0.146).

### 3.4 Non-paternity

Increasing the rate of non-paternity drew the putative father's association towards the mother's. For example, with non-paternity assumed at 15% the association between paternal height and growth in length from 29 weeks to birth changed from 0.13SD (per SD increase in father's height) to 0.16SD, the mother's coefficient reduced by only 0.004SD from 0.23SD. However, the effect of non-paternity was very small when the putative father's association was close to zero. Thus the patterns where there is a suggestion of an association for the mother but no clear evidence for the father, for example the associations with placental size, were unaffected by rates of non-paternity.

## 4 Discussion

To the best of our knowledge, this is the first study to examine parental height and BMI associations with ultrasound measures of fetal growth in a rural Indian population with a high prevalence of low birth weight. Our results suggest that associations between paternal height and fetal head circumference and femur length are detectible earlier in gestation (17–29 weeks) compared to the same associations with maternal height. Fetuses of mothers with a higher BMI had smaller head circumferences at 17 weeks, but head growth caught up to become larger in late gestation. Paternal BMI showed no significant associations with any component of fetal growth. Maternal but not paternal BMI, was positively related to placental size at 17 weeks.

Our study has several important strengths. First, we used serial fetal biometry to characterise prenatal growth as opposed to birth weight. Second, ultrasound measurements were made by only 2 sonographers and intra and inter-observer reliability was excellent. Third, we were able to obtain accurate LMP dates since health workers visited women every month prior to pregnancy. Fourth, the maternal BMI data are likely to be an accurate representation of pre-pregnant BMI as data were collected within a 3 month window prior to pregnancy. Fifth, a sensitivity analysis showed that non-paternity was unlikely to have affected our main findings. And last, the use of conditional growth variables as a model of fetal growth allowed an exploration of the onset and pattern of parent–fetal associations.

The main limitation is a lack of power, a bigger study may have exposed some of the trends in the timing of associations over gestation that we reported, however intergenerational data with fetal ultrasound from populations in rural India are rare. We were also restricted to investigating growth intervals at relatively fixed time points. However, while more densely spaced measures may have offered a deeper insight, it does increase signal-noise ratio in the growth variable.

Maternal height was positively related to conditional growth in the last gestational interval (29 weeks — birth) for head circumference and femur/birth length. A study of American singletons also found positive associations between maternal height and head circumference at 31 weeks, and with femur length at 25 weeks [14]. Paternal height was positively related to conditional head circumference growth from 17 to 29 weeks and size of the femur at 29 weeks. The associations with father's height were thus evident earlier in gestation compared to the associations with maternal height. In an Australian study, paternal height was positively related to fetal femur length at 24 weeks, while maternal height was negatively related [27]. We could find no other studies to compare this differential pattern.

Positive correlations of maternal and paternal height with newborn skeletal measurements like length and head circumference are well described [6]. Our new finding that correlations between paternal height and fetal size and growth occurred earlier in gestation compared to associations with maternal height, may be related to the strong correlation between paternal height, but not maternal height, and placental volume at 17 weeks gestation. It suggests that paternally inherited genes influencing skeletal growth are expressed throughout gestation, while those from the mother are expressed in late gestation, and may be related to larger placental size. These differences could be mediated by epigenetic mechanisms; it is known that a number of genes that promote fetal growth are paternally imprinted [28,29]. There is insufficient data like ours to know if the associations observed are specific to our population or seen in other populations. Taken together, the maternal and paternal findings would be consistent with the ‘selfish gene’ theory, which suggests that paternally inherited genes promote fetal growth, regardless of maternal nutritional status [30].

For maternal BMI, there was a positive association with HC growth in the 29 week to birth interval, consistent with findings from Goldenberg et al. (1993) [14], who found positive associations with HC at 31, 36 weeks and birth. The associations with maternal BMI at 17 weeks were negative for all fetal components in our study. In particular, in this population with a high prevalence of chronic maternal undernutrition, women with lower BMI had fetuses with larger head circumference at 17 weeks. A comparison of our finding with the American study [14] is not possible because the coefficients were not reported, however, Blake et al. [27] did report negative associations between femur length and both maternal and paternal BMI, although the evidence was weak. It is unlikely that our results are due to gestational dating errors related to maternal BMI, as there was no relationship between the discrepancy in ultrasound and LMP dated gestational age and maternal BMI (p = 0.6), unlike that found in another study [31]. Pre-pregnancy BMI is a marker of energy balance, nutrition and adiposity. Interestingly, maternal BMI was positively associated with placental volume at 17 weeks. An explanation for this differing placental–fetal response could be that better nutrition (higher maternal BMI) stimulates placental development at the expense of the fetus in early pregnancy, possibly as a strategy to enable greater fetal growth in late gestation. A larger placenta, whilst requiring more energy itself, has a larger surface area, which allows increased nutrient transfer to the fetus.

The different patterning of associations reflected by the mother and father's BMI on placental volume and the suggestion of a different pattern of associations on the fetal components — particularly head circumference, suggest some direct and potentially modifiable effects of maternal nutritional status on placental and fetal growth. Higher maternal BMI may promote placental growth in early-mid pregnancy, and this may enhance fetal growth in late pregnancy, although this interpretation of our findings is speculative. We do not yet know the implications of variations in placental and fetal growth for future health in the child. Recent studies in the Helsinki birth cohort suggest that placental size and shape at birth, newborn size, and ratios of placental to newborn size, predict adult hypertension, and that these associations are conditioned by maternal nutritional status [32]. In further follow-up of this cohort, we will be able to explore associations of placental measurements and fetal growth patterns with cardio-metabolic risk factors in the children.

We recommend that epidemiological studies exploit the use of ultrasound to characterise variations in fetal and placental growth, and their timing during gestation. Such measures will allow more carefully designed analyses into questions related to the developmental origins of disease, and offer insight beyond that possible with birth weight alone.

## Figures and Tables

**Table 1:** Characteristics of the study sample.

	n	Mean (SD)
Mother		
Age (years)^a^	557	21 (19, 23)
Height (m)	557	1.52 (0.05)
Weight (kg)	553	41.8 (5.1)
BMI (kg/m^2^)^a^	553	17.9 (16.7, 19.1)
Nulliparous (n, %)	557	177 (31.8)
Father		
Age (years)^a^	222	28 (25, 31)
Height (cm)	532	1.65 (0.06)
Weight (kg)	537	52.8 (7.9)
BMI (kg m^2^)^a^	532	19.0 (17.6, 20.7)
Baby (at birth)		
Female (n, %)	557	259 (46.5)
Weight (g)	519	2658 (361)
Length (cm)	538	47.7 (2.0)
Head circumference (cm)^a^	539	33 (33.2, 34.0)
Abdominal circumference (cm)^a^	539	28.6 (27.5, 29.8)
Gestational age (weeks)	557	39.5 (1.2)
Religion (n, %)		
Hindu	557	538 (96.6)
Muslim		16 (2.9)
Buddhist		3 (0.5)
Household Smokers (n, %)^b^	557	141 (25.3)

a Median (IQR).

b All smokers were male.
